# Respiratory Complications of Adenotonsillectomy for Obstructive Sleep Apnea in the Pediatric Population

**DOI:** 10.1155/2018/1968985

**Published:** 2018-11-01

**Authors:** G. Marrugo Pardo, L. F. Romero Moreno, P. Beltrán Erazo, C. Villalobos Aguirre

**Affiliations:** ^1^Titular Professor, Department of Otorhinolaryngology, Universidad Nacional de Colombia, Bogotá, Colombia; ^2^Chief of Pediatric Otolaryngology Department, Fundación Hospital de la Misericordia, Bogotá, Colombia; ^3^Department of Otorhinolarynglogy, Universidad Nacional de Colombia, Bogotá, Colombia

## Abstract

**Objective:**

To determine the prevalence of respiratory complications in the early postoperative period of children with sleep apnea who required adenotonsillectomy at a tertiary pediatric hospital and to establish recommendations for postoperative monitoring.

**Methods:**

Retrospective cohort study of children with obstructive sleep apnea (OSA) diagnosed by polysomnogram (PSG), who underwent adenotonsillectomy for treatment of OSA. The prevalence of respiratory complications in the first 24 postoperative hours was measured. Patients with craniofacial malformations, obesity, and severe cardiovascular comorbidities were excluded. The prevalence of postoperative respiratory complications was compared with the severity of OSA according to the Apnea Hypopnea Index (AHI) and NADIR. All data were taken in patients residing in Bogotá city, Colombia, at 2.640 meters above sea level (m.a.s.l).

**Results:**

Between May 2014 and February 2017, 167 patients (108 males) required adenotonsillectomy for OSA, with an age range of 1 and 15 years (mean 5.3 years +/- 2.7). The prevalence of postoperative respiratory complications was 3.59% (6/167). There was a statistically significant relationship between the presence of respiratory complication and AHI greater than 44/h (p <0.04). There was an inverse correlation between the AHI and NADIR values. Risk groups of patients younger than 3 years and NADIR less than 70% had a higher prevalence of respiratory complications; however, this correlation was not statistically significant (p <0.08 and 0.89, respectively).

**Conclusions:**

The prevalence of respiratory complications in OSA patients undergoing adenotonsillectomy in high altitudes is similar to that reported in other heights. Preoperative AHI greater than 44/h could be considered a risk factor for early respiratory complication. We suggest ambulatory management after 6 hours in Postanesthetic Care Unit (PACU) observation in patients older than 3 years, with AHI less than 44/h and NADIR greater than 70% in altitudes higher than 2.500 m.a.s.l. Further research must be done to confirm this hypothesis.

## 1. Introduction

In 2006, the American Society of Anesthesiologists (ASA) published the guidelines for the perioperative management of patients with OSA with special emphasis on risk factors like obesity and age under 3 years [1]. In most studies the average number of apneas and hypopneas per sleep hour (AHI) and the lowest value of oxygen saturation during sleep (NADIR) have been used to evaluate severity of OSA and treatment outcomes [1–3]. In 2012 the American Academy of Pediatrics published guidelines with recommendations on the intrahospital postoperative care of patients with severe OSA (AHI>24/H, NADIR <80%, or PCO2 >60 mmHg in PSG), children under 3 years, and children with severe comorbidities or obesity [3]. In 2014, the ASA guidelines update report recommended that patients under 3 years, with severe OSA (AHI >10/h) or NADIR <80% or other comorbidities should be admitted to a critical or intermediate care unit in the early postoperative period [4]. There is still conflicting evidence regarding the recommendation for elective entry of patients in the postoperative care units in altitudes higher than 2.500 meters above sea level (m.a.s.l) [5, 6].

Postoperative respiratory complications in adenotonsillectomy surgery can vary between 1.4 and 5% [7]. Major ones include pulmonary edema, laryngospasm, and bronchospasm. Minor complications include hypoxemia, hypercapnia, and apnea exacerbation events [8]. There are a number of identified risk factors for postoperative respiratory complications; however, none of these studies were done in physiological oxygen deprivation. Eight percent of the world's population resides above 1.600 m.a.s.l, and only two percent resides above 2.500 meters, including Bogotá [9]. At this altitude, the relative hypoventilation that accompanies sleep onset may induce a significant drop in Spo_2_ (peripheral capillary oxygen saturation) and exacerbate desaturation values; however it has not been proven yet that the respiratory complications rate is higher compared with other heights [6, 9]. This is the first study made about respiratory complications in OSA patients in high altitudes.

Our main objective is to determine the prevalence of respiratory complications in children requiring adenotonsillectomy for sleep apnea in the early postoperative phase and to establish a recommendation for postoperative monitoring and safety hospital discharge.

## 2. Materials and Methods

A retrospective cohort study was performed on children with OSA diagnosed by PSG, who were admitted to* Fundación Hospital de la Misericordia *in Bogotá, Colombia, between May 2014 and February 2017, and in whom tonsillectomy was performed (either with or without adenoidectomy). Patients with a diagnosis different to OSA, incomplete data in clinical record, or BMI Z-score > 2 or in the 95th percentile were excluded. Children with craniofacial malformations or other associated cardiovascular comorbidity were excluded.

### 2.1. Polysomnography and Postoperative Monitoring

Standard in-laboratory PSG was performed with an attending sleep technician, with indication of high suspicion sleep apnea based on a clinical score. An accredited laboratory performed the exam with the interpretation of a pediatric physician board, certified/eligible in sleep medicine. Measured parameters included bilateral electrooculography, electroencephalography, electromyography, continuous airflow monitoring with a nasal pressure transducer, oxygen saturation using a pulse oximeter, and end tidal carbon dioxide using a CO2 sensor and chest and abdominal effort. Obstructive apnea and hypopnea were defined based on AASM guidelines [10]. Postoperative monitoring in the PACU and intermediate care unit (IMCU) includes pulse oximetry measurement, capnography, and continuous nurse observation.

### 2.2. Intervention

Tonsillar and adenoid hypertrophy was diagnosed with physical examination and fiber optic nasopharyngoscopy (using a 2.7-mm, 0-degree telescope, Karl Storz, Germany). The Brodsky grading scale was used to determine tonsil size [11] and Parikh classification for adenoid size [12]. Weight-for-age Z-scores were calculated according to the WHO/National Center for Health Statistics reference [13]. Clinical sleep apnea assessment includes snoring (never, occasionally, and frequently), apnea (absent or present), and difficulty breathing during sleep. The prevalence of respiratory complications was measured during patients stay in the IMCU in the course of early postoperative recovery in the first 24 hours.

### 2.3. Statistical Analysis

The data for postoperative respiratory complications was compared with the data from the PSG, specifically the AHI and the NADIR, using Fisher's Test and univariate logistic regression analysis. Descriptive statistics, including means and standard deviations for continuous variables and percentages for categorical variables, were used to compare the outcomes groups. Chi-square analysis was used to compare categorical data between groups. The Rho Spearman coefficient was calculated to establish the relationship between the variables corresponding to AHI and NADIR. Statistical significance was considered with p values less than 0.05. This investigation was previously accepted by the medical ethical committee from* Fundación Hospital de la Misericordia* and the identity of patients was adequately protected thought the study.

## 3. Results

**Figure 1:**
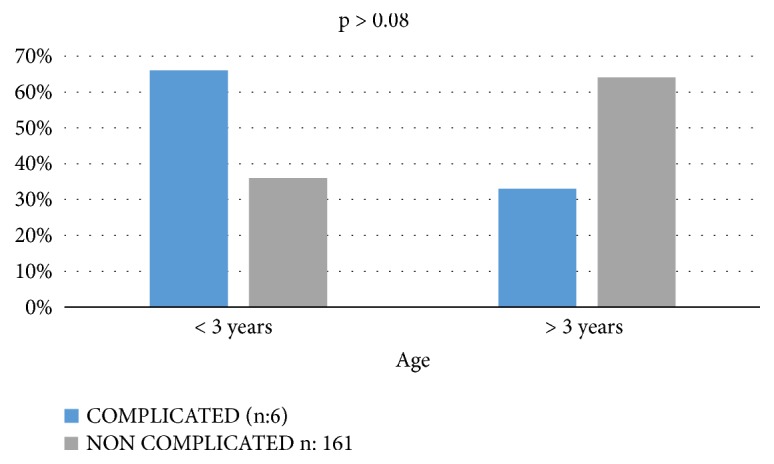
**Comparison between groups of age**. Patients younger than 3 years, compared to patients older than 3 years, had a higher incidence respiratory complications in the immediate and early postoperative care. There was no significant difference between both groups (p > 0.08).

A total of 167 patients were taken to adenotonsillectomy, with a median age of 5.3 years+/- 2.79 years, 109 boys and 58 girls. The population characteristics are presented in Table 1. There were no significant differences in age, gender, or baseline adenoids-tonsil size between groups. The prevalence of postoperative respiratory complications was 3.59% (6/167). The average AHI of the complicated and noncomplicated group was 43.9/h and 29.9/h, respectively. We approximated the first value to 44 and considered it as the numeric boundary for determining the risk of complication. Statistical significance was found between the presence of respiratory complications and a preoperative AHI greater than or equal to 44/h, p < 0.04 (95% confidence interval (CI): (-28.91) - (-0.62)). The group of patients younger than 3 years, compared to patients older than 3 years, had a higher incidence of respiratory complications in the immediate and early postoperative phase (Figure 1). An association was also documented between patients with a preoperative NADIR below 70% and postoperative respiratory complications; however, for all of them, no statistical significance was found (p value < 0.08 and p< 0.89, respectively (Table 2)).

**Figure 2:**
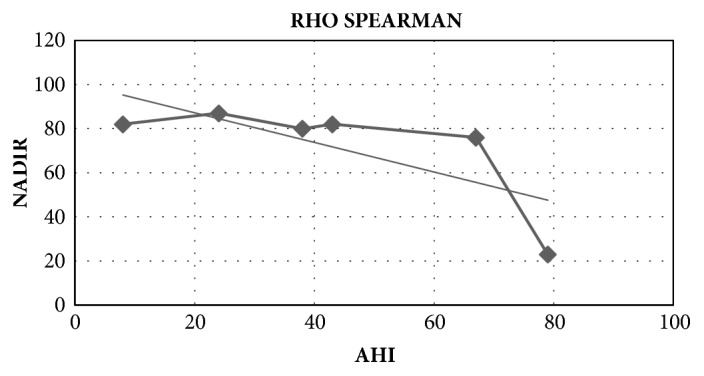
**NADIR versus AHI. Relationship: Rho Spearman coefficient**. An inverse correlation was documented between the data for AHI and NADIR, according to the Rho Spearman coefficient (-0.36).

An inverse correlation was documented between the data for the AHI and NADIR, according to the Rho Spearman coefficient (-0.36), which suggests that as values for the AHI increase, the value of minimal oxygen NADIR tends to diminish (Figure 2). Eighty-five percent (143/167) of patients required postoperative monitorization and clinical surveillance in IMCU, given the diagnosis of severe OSA. Nonetheless only 3.4% (5/143) of severe OSA patients had some respiratory major complications: 2 had bronchospasm, 2 had laryngospasm reflex, and 1 patient needed reintubation. Only one minor complication, severe hypoxemia, was identified in a mild OSA patient who was treated with supplementary oxygen titration (Table 3). The laryngospasm cases were successfully treated with positive pressure ventilation. While the two bronchospasm cases received an inhaled B2 agonist at low doses and systemic corticosteroid, with adequate response to treatment. The reintubation case occurred in the immediate postoperative time in a 7-year-old patient, with residual muscular relaxation. The patient was successfully extubated in the following hours, without further complications. Only 2.9 % (5/167) of patients required prolonged hospitalization due to optimization of pain management and oral intolerance.

## 4. Discussion

Obstructive sleep apnea is defined as a prolonged partial obstruction of the superior respiratory airway or the complete intermittent obstruction that alters the normal ventilatory pattern during sleep [1–3]. It is estimated to affect 1.2 to 5.7% of the pediatric population worldwide and the diagnostic gold standard is the PSG [4]. Apnea in the pediatric population is defined as a respiratory pause over two respiratory cycles, while hypopnea is defined as an airflow reduction of 50% or more associated with awakening or desaturation greater than 3% of baseline [4]. Currently, in children younger than 14 years it is considered pathological if AHI is equal to or greater than 1/h. OSA severity is classified according to the AHI value as follows: mild 1.5 to 4.9/h, moderate 5 to 9.9/h, and severe more than 10/h [3, 4, 6]. In 2011, the most frequent OSA treatment in the childhood was adenotonsillectomy, performed in nearly 530,000 children younger than 15 years in the United States [7].

In 2014, del-Río, Camacho et al. published a study evaluating the need for postoperative IMCU in patients undergoing adenotonsillectomy for severe OSA, in which the rate of respiratory complications was 2.2%, without demonstrating statistical significance among the severe OSA group compared to patients with adenotonsillectomy for other reasons. All complications occurred during the immediate postoperative period [14]. Keamy et al. published in 2015 a study identifying predictive factors for complications after adenotonsillectomy in children with severe OSA. They found AHI > 15/H and NADIR <80% as strong predictors for respiratory complications [15]. Furthermore, NADIR < 80% was identified as the most important factor associated with increased oxygen requirements, longer hospital stay, and the incidence of complications after discharge from postanesthesia care units. These findings suggest that patients with those characteristics could benefit from postoperative recovery in PACU or a more strict surveillance after surgery [15, 16].

Theilhaber et al. published in 2014 their experience regarding the frequency of postoperative complications after adenotonsillectomy in children with OSA, where adverse events occurred in 36% of population, with an average time to presentation of 156 minutes. The negative predictive value for a nonmajor to mild adverse event in the postoperative period, after recovery in the PACU without complications, was 98.3%. They suggest that routine surveillance in the ICU during postoperative recovery is unnecessary. They advocate extended surveillance in the postanesthesia unit if possible, reserving IMCU admission to those patients with additional high risk factors [17].

In 2015, Duenas et al. [9] reported that pulse oximetry values, just like AHI, vary according to height above sea level. In Bogotá, as well as every other city located above 2.500 meters, cut points to distinguish pathological from physiological data in sleep apnea during PSG could be higher than other populations, so the protocols and clinical outcomes in sleep apnea patients should be established according to local data and should not be adapted from parameters determined in studies made at sea level. Sleep disorders in height should be discussed separately by interdisciplinary medical groups [5, 6, 18].

The results of the present study show a percentage of respiratory complications close to 4%, very similar to data reported in medical literature [19, 20]. From those patients who had major complications, 80% had preoperative AHI values greater than 40/h, strongly superior to the total average. The logistic regression analysis suggests an AHI equal to or greater than 44/ h, as a possible predictive value for postoperative respiratory complication in patients living at 2.640 m.a.s.l. Children younger than 3 years or with NADIR value under 70%, may have a higher risk for complications; however this association did not show statistical significance. No correlation was found between the different types of complications and the demographic characteristics in the population included. We try to exclude all the known comorbidities and possible variables that would affect the postoperative complication rate. Gender, BMI, and adenoids/tonsil size did not affect the respiratory outcomes in healthy patients.

Although no follow-up was performed during the late postoperative period, a database search was conducted in the hospital's emergency consult system during the study and no readmission for late postoperative complications was identified. Postoperative PSG data were found in only 50% of patients in complicated group; in all of them, AHI and NADIR values improve considerably after surgery.

This is the first study that measures the postsurgery complication rate in high altitudes. The analysis confirms a similar prevalence of major respiratory events compared to values reported in other heights [19, 21, 22]. The authors suggest preoperative AHI value of 44/h as a cut point for prediction of respiratory complications in high altitudes (> 2.500 m.a.s.l). Patients older than 3 years, without risk factors or associated comorbidities, adequate family support, nearby residence, and preoperative values of AHI under 44/h and NADIR over 70%, could undergo postoperative monitorization in the PACU with hospital discharge in the first 6 hours after surgery. These recommendations look forward to decreasing occupancy of the IMCU, to optimize the operating room surgical schedule and to improve the perioperative care in OSA patients.

## 5. Conclusion

The prevalence of respiratory complications in the early postoperative period of adenotonsillectomy in OSA patients in high altitudes is similar to that reported at sea level. Preoperative AHI greater than 44/h could be considered a risk factor for early respiratory complication. We suggest ambulatory management after 6 hours in PACU observation in patients older than 3 years, without comorbidities with adequate preoperative PSG values. The perioperative and postoperative care in OSA patients should be standardized in all health institutions located at high altitudes.

## 6. Study Limitations

Further studies with greater evidence and more population are needed to verify our findings. Despite the small number of complicated cases, this sample size reached statistically significant differences. Probably a larger sample size will show even greater differences.

## Figures and Tables

**Table 1:** Demographic characterization of all patients undergoing adenotonsillectomy for OSA indication.

**VARIABLE**	**CHARACTERISTIC**	**TOTAL**	**MEAN/** **PERCENTAGE**
**Age**	Range 1-15 years	167	5.3 (+/- 2.79)*∗*
	< 3 years	57	33%
	> 3 years	110	67%

**Gender**	Male	109	65%
	Female	58	35%

**OSA Severity**	Mild	6	4%
	Moderate	18	10%
	Severe	143	86%

**Adenoids Hypertrophy Grading system (Parikh)**	Grades I-II	29	17%
	Grades III - IV	138	83%

**Tonsillar Hypertrophy Grading system (Brodsky)**	*∗∗*Grade II	21	12%
	Grade III	103	62%
	Grade IV	43	26%

**Weight-for-age Z-scores (WHO classification)**	<-2	1	0.5%
	-2 to – 1	6	3.5%
	-1 to +1	126	75%
	+1 to +2	35	21%

**Time of Complications**	Total	6/167	3.59%
	Immediate (<12 h)	4/6	66%
	Early (>12 h)	2/6	34%

*∗*Standard deviation. *∗∗*No patients with tonsil grade I were found.

**Table 2:** Patient's characterization according to risk groups.

**VARIABLE**	**COMPLICATED**	**NON COMPLICATED**	**P VALUE**
	**n:6**	**n: 161**	
**Mean age, years (SD)**	3.2 (2.1)	4.5 (2.9)	0.12

**Subgroup > 3 years**	2 (33%)	108 (64%)	0.08
**Subgroup < 3 years**	4 (66%)	53 (36%)	

**Mean BMI**	16.15	15.9	0.15

**Preoperative AHI**	43.90	29.96	**0.04** **∗**

**Preoperative NADIR**	70%	76.38%	0.89

SD: standard deviation. *∗*95% confidence interval (CI): (-28.91) - (-0.62).

**Table 3:** **A.** Time to presentation of respiratory complication in the initial 24 hours after surgery; **B.** preoperative AHI and Nadir values in the complicated group.

**TYPE OF COMPLICATION**		**TIME TO PRESENTATION **	**B. Pre AHI**	**Pre NADIR**
*Major*	**Laryngospasm (2)**			
	**Patient #1**	0 – 6 hours	53.5	58%
	**Patient #2**	0 – 6 hours	29.0	62%
	**Bronchospasm (2)**			
	**Patient # 3**	12 – 24 hours	69.6	87%
	**Patient #4**	6- 12 hours	49.9	78%
	**Reintubation (1)**			
	**Patient #5**	0 – 6 hours	52.5	69%

*Minor*	**Severe hypoxemia (1)**			
	**Patient # 6**	0 – 6 hours	8.9	66%

## Data Availability

The data used to support the findings of this study are included within the article and could be provided from the corresponding author upon request.
